# Advice to a Young Mathematical Biologist

**DOI:** 10.1007/s11538-024-01269-1

**Published:** 2024-04-09

**Authors:** Paul A. Roberts

**Affiliations:** Centre for Systems Modelling and Quantitative Biomedicine, Institute of Biomedical Research, University of Birmingham, Birmingham, B15 2TT UK

**Keywords:** Career, Funding applications, Job applications, Mentorship, Networking, Teaching and research

## Abstract

This paper offers advice to early-mid career researchers in Mathematical Biology from ten past and current Presidents of the Society for Mathematical Biology. The topics covered include deciding if a career in academia is right for you; finding and working with a mentor; building collaborations and working with those from other disciplines; formulating a research question; writing a paper; reviewing papers; networking; writing fellowship or grant proposals; applying for faculty positions; and preparing and giving lectures. While written with mathematical biologists in mind, it is hoped that this paper will be of use to early and mid career researchers across the mathematical, physical and life sciences, as they embark on careers in these disciplines.

## Introduction

Early-mid career researchers in Mathematical Biology face a particular set of challenges. As they develop in their career, a number of skills need to be learnt, most of which are not taught in a typical undergraduate degree. In this paper, ten leading mathematical biologists—all current or former Presidents of the Society for Mathematical Biology (SMB)—share their advice on a number of areas of particular interest to early and mid career researchers. While written with mathematical biologists in mind, much of the advice presented here is of relevance to any researcher working in the life, physical or mathematical sciences. It is hoped that this paper will prove a valuable resource to early and mid career researchers as they make the first steps in their academic journey, providing a helping hand from those who have trodden the road before them.

The idea for this paper occurred to me following the excellent past Presidents’ panel discussion, organised by Prof. Heiko Enderling, at the 2023 SMB conference, held at The Ohio State University in Columbus, Ohio. This was an inspiring session, with many useful insights shared by some of the greats in the field. It struck me that it would be good to capture the insights from some of these researchers in a permanent way, and that this would be of particular interest and benefit to early/mid career researchers.

All of the living past and current SMB Presidents were contacted, and to those who were able to contribute, a series of questions was posed, inviting their top tips and advice in a number of areas relevant to early/mid career researchers. These questions consisted of a subset of ten specific topics, together with two, more general questions, which were posed to all contributors. Responses were then compiled, ordered and edited to provide coherent guidance in each area.

The advice offered here is not intended to be exhaustive. Rather, it is hoped that this will be a starting point, bringing together guidance on a range of topics into a single place, leaving the reader to explore specific areas in greater depth as desired. As with any advice, it is left to the reader to follow or leave at their discretion.

The title of this article is a homage to Prof. Sir Peter Medawar’s book ‘Advice To A Young Scientist’ (Medawar, 1979) and to the later multi-author chapter ‘Advice to a Young Mathematician’ in The Princeton Companion to Mathematics (Atiyah et al., 2008); both of which are recommended. To the best of my knowledge, this is the first paper to offer guidance specifically to early/mid career mathematical biologists.

In what follows, we cover ten specific topics: ‘Deciding if a career in academia is right for you’, ‘Finding and working with a mentor’, ‘Building collaborations and working with those from other disciplines’, ‘Formulating a research question’, ‘Writing a paper’, ‘Reviewing papers’, ‘Networking’, ‘Writing fellowship or grant proposals’, ‘Applying for faculty positions’ and ‘Preparing and giving lectures’; together with two general topics: ‘What do you wish you had known when you were an early-mid career researcher?’ and ‘Some final words of advice’. These sections can be read in any order or in isolation, depending on the needs and interests of the reader.

## Deciding if a Career in Academia is Right for You

Many of us may wonder if the academic path is the right one for us. This question might occur when deciding whether or not to pursue a doctorate, to apply for postdoctoral or faculty positions, or even whether to remain in academia, having obtained a permanent position. Whatever your stage, the following advice may be helpful to bear in mind.

- Make a list of things that are important to you, what you want to accomplish in your professional life and what will make you happy going to work every day for the rest of your life.
- If you are self-driven, have lots of questions, like to work and meet with people, and like to share your work in different venues (e.g. papers and presentations), you could consider a career in academia.
- A career in academia is not easy.
- You need to consider what kind of academic you would like to be: more research focused, or more teaching focused. Do you want to have a large or small group, or work at a large or small school?
- You also need to consider that there is a lot more to an academic job than what you may have experienced during your undergrad/PhD/postdoc. Talk to PhD students, postdoctoral fellows and faculty to find out what they do from day to day, and get a sense of what the job entails. Learn what they like about their roles and what they wish was different.
- Talk to many professionals outside academia about their experiences.

## Finding and Working with a Mentor

The concept of a mentor is a familiar one, both historically and in popular culture: Plato had Socrates, Luke Skywalker had Obi-Wan Kenobi, Bertrand Russell had Alfred North Whitehead and Frodo had Gandalf. Though familiar, finding and developing such a relationship can be difficult. Here are some expert tips on how to navigate this area.

- The mentor is probably the most important part of your academic career.

**Finding a mentor**

- You can find a mentor in many different ways:
  - Get to know the faculty in your research institution;
  - Talk to people at conferences;
  - Participate in mentoring programs.
- Have a one-on-one conversation about ideas and what the potential mentor looks for.
- Identify what YOU need from a mentor. Make sure that you communicate your needs to a prospective mentor and evaluate if they can help you in your academic journey.
- Be honest about your interests.
- The best science is not necessarily done by the mentor that best serves your needs, though make sure the research approach of the mentor excites you.
- Try to visit and meet members of the potential mentor’s group, talking with former/current students/collaborators/mentees. This is important, not least because it will enable you to check the potential mentor’s reputation. This will also help you to evaluate if their mentorship style is right for you.
- Take into account the breadth of the institution, and especially the department, and the potential to interact with others outside the group.

**Working with a mentor**

- Expect the relationship to develop and change over time.
- Mentorship can be developed very naturally—through discussion at conferences and workshops, and then some emails in between.
- Make time for the relationship to develop in social contexts in connection with or outside of research discussions (e.g. coffee/tea/beer etc. time).
- Do not agree to work on a project if it does not align with your interests, but be open to suggestions of new projects or research questions/approaches.

## Building Collaborations and Working with Those from Other Disciplines

Given the intrinsic interdisciplinarity of mathematical biology, the ability to build and grow fruitful collaborations is key to developing biologically faithful and impactful models. This is not something that is usually taught at the undergraduate level, but rather is learned on-the-job, with a degree of trial-and-error(/-improvement). While this is a rite-of-passage that all mathematical biologists must pass through—and, indeed, a lifelong learning process—here are few tips to smooth the way.

- Listen carefully to lectures on topics from other disciplines, and read review papers carefully to identify what questions motivate that discipline/topic. Ask yourself in what way you could contribute to answering such questions using your skill-set.
- Learn a lot about the subject matter. Attend experiments when they are being done.
- Follow your heart and make the effort to work with people who you find interesting and exciting.
- Find someone who is open to theoretical approaches and who is a person with whom you get along really well.
- It can take a while to build a good collaboration, so be patient, and invest in a few possible directions. Usually one or another will eventually pan out.
- Trust that your collaborators know what they are talking about.
- Ask a lot of stupid questions, balancing keeping expectations low with occasional moments of surprising brilliance.
- Be clear about shared responsibilities.
- Be willing to suppress your ego. Remember that what makes the work interesting is the experiments rather than the theory.
- Learn the jargon of the biological discipline(s) relevant to your research.
- Explain your ideas in plain English. Do not expect potential collaborators to know or be familiar with mathematical jargon or methods.
- Explain what your methods could do to help test hypotheses or to analyse data, or to help with the design of experiments.
- Try to get in a situation where you can help design the experiments to provide data needed for analysis.
- Biological experiments usually cost a lot of money and take a lot of time. Do not expect that a collaborator will immediately agree to do your favourite experiment. (Sometimes, you have to make-do with data from the literature.)
- Be willing to pay any students who may work on the theory and perhaps other costs associated with doing the experiments. Working on joint grants is one way to do this but that takes patience.

## Formulating a Research Question

As any Douglas Adams fan will know, the key to making discoveries lies in asking the right questions. The following advice may be helpful in deciding upon a research topic and what question(s) to ask.

- Find a problem that really interest you, about which you are passionate and want to know the answer, and do not care what others think.
- Be driven by the research question, not by the methods you will use.
- Find a topic that will potentially expand the field, not something that is just incremental.
- There are many kinds of research questions: explaining a puzzling data set; testing a hypothesis for some mechanism; finding some optimal strategy; making a long-term prediction. Each case would imply a different strategy.
- To find new interesting quantitative questions, read a number of recent review papers on the topic of your choice. Find sentences such as ‘The mechanism for this observed behaviour is poorly understood’, and look for key areas where a knowledge-gap is identified. Be sure that these questions are not just experimental ones. Be sure that some facts are known and/or some data is available on which to construct your model, for example.
- Be open to approach by colleagues from other disciplines. Listen to their ideas and motivation, and assess whether your skills could be useful, or whether other colleagues have just the right tools to be helpful.

## Writing a Paper

Most mathematical biologists begin by taking an undergraduate degree in mathematics, spending the bulk of their time working through a series of problem sheets. As such, when they come to do a doctorate and begin writing their first paper, it may be some years since they were required to write at any length. Further, the process of writing an academic article is unlike that of writing a secondary/high school essay. The following advice should be of help in providing a possible approach to writing papers, while also highlighting some common pitfalls.

**Some general points**

- Do your literature review well: you do not want to submit a manuscript that is missing important references.
- Spend time critically reviewing your results. Do they make sense? What are some questions that reviewers might have? Are any results difficult to understand?
- Do not make the paper too long. Figure out what you want to say in a direct way.

**A possible approach**

Let us assume you have wrapped up an original piece of research and you are ready to write your first paper. The first step is to get your work organized in a logical, convincing fashion. You have probably already done this in preparing for your committee meetings, student presentations and poster sessions. A good MS PowerPoint presentation is a great place to start.

Next, consider the audience you want to reach. Defining your audience will dictate what journal to submit to and also what background information you need to include in the introduction.

Write an outline, using the standard format of a scientific publication.

- Title: start with a working title; it may change later.
- Abstract: write this last!
- Introduction: make an outline, with your target audience in mind.
- Results:
  - arrange your research in a logical fashion;
  - sketch your figures in some detail (and write cogent legends);
  - consider what tables you will need;
  - push some results to ‘Supplementary Material’ to stress the main points.
- Discussion: make notes along the way, but write this part later.

Now that you are ready to start writing, keep the following **Four Cs** in mind.

1. **Correct.** Everything you write must be scientifically correct, to the best of your knowledge. Check each sentence and every equation. Make sure you have provided the correct parameter values for all your calculations.
2. **Clear.** Now that everything is correct, you must communicate your results clearly to your audience. You do not have to tell people what DNA means, but do not skip over important things that the reader needs to know. It is helpful here to get someone else’s point of view—on joint authored papers, it is the responsibility of all authors to make sure that what is written is clear. Some important points to note:
  Often papers are not structured in a logical way, and read like a stream of consciousness. Look at the logical structure of your flow of ideas to make sure that your argument will make sense to your readers.
  In this regard, basic grammar rules are important, especially coherent paragraphs with topical sentences. Do not let your paragraphs get too long; most long paragraphs can be broken into two or more separate ideas.
  Watch how you use pronouns—they can be dangerous. You may know what your pronoun is referring to, but your reader may not. When a reader comes across a pronoun, he/she typically assumes that the pronoun refers to the last noun mentioned in the previous sentence. If the reader has to look further back, he/she will likely get lost. The simple fix is to repeat the noun, so it is absolutely clear what you are talking about.
  Another mistake of non-English writers is overloading the subject of a sentence, using too many modifiers for a noun, or other nouns as modifiers of the main noun. It can be difficult for the reader to figure out what the noun of the sentence is, and which words are modifiers. The simple fix is to use prepositional phrases and dependent clauses to expand on a noun, rather than going beyond a few adjectives. For example, ‘the budding yeast cell cycle spindle assembly checkpoint’ should be ‘the spindle assembly checkpoint of the budding-yeast cell cycle’. Another good example of an ‘overloaded noun’ of a sentence is: ‘Initiation and progression of the cell cycle are considered to occur in response to *the timely ordered transcriptional, post-transcriptional, and posttranslational regulation of the cell cycle (cyclin/cyclin dependent kinase [CDK]) machinery components*’. The italicised phrase is the object of the passive verb construction ‘are considered to occur in response to’. The object is ‘components’ and the preceding words all modify ‘components’. It would be clearer to write: ‘Progression through the cell cycle is thought to be based on the temporally ordered activation of cyclin/cyclin dependent kinases (CDKs), which are regulated by a complex molecular network of transcriptional, post-transcriptional, and post-translational controls’.
3. **Concise.** After you are sure your text is correct and clear, then go through it carefully to get rid of annoying repetitions that may have crept in. Pare things down to a minimum without destroying clarity. State your main points several times (in the Abstract, Results and Discussion); as for everything else, just say it once.
4. **Compelling.** Finally, polish up the writing. Use MS Word’s thesaurus to find exactly the right word to get your idea across. Make the paper easy/pleasant/attractive to read, so people will recommend it to others.

## Reviewing Papers

Reviewing your first paper can feel like a daunting task, with a weight of responsibility to make an accurate and fair assessment. The following tips should prove useful both to first time reviewers, and to those with some experience under their belts.

- Only accept reviews for manuscripts you are competent to assess.
- Make sure you are familiar with other research in the field, so you know how novel the work is.
- Do not take on another review if you already have one.
- Negotiate with the editor a timeline that suits you and not just them.
- Do not allow deadlines to make you do a superficial job.
- Try to be fair and write the kind of review you would like to receive.
- Read the introduction and discussion first, to get a feel for what the authors want you to get from the paper, then read the whole manuscript to see if the results match with this.
- Do not question the motives but focus on the results.
- Do not be sucked in by overhype.
- Always ask for codes to be shared if they are not already.

## Networking

Our scientific research is not conducted in isolation, but rather as part of a community. As such, developing relationships with fellow scientists and mathematicians is an important part of any mathematical biologist’s career. Indeed, the contacts we make now could be our future collaborators, reviewers or employers. We often use the word ‘networking’ to denote the practice of making and developing these relationships, particularly in the context of conferences. While most would agree that networking is important, many of us are unsure of how best to go about it. This problem is especially acute for early and mid career researchers, who may wish to speak with senior researchers, but are unsure of how to introduce themselves, or manage the conversation. Here is some advice on how to approach it.

- Study the conference program before the meeting. Identify 4–6 people with whom you might be interested in meeting. These include people that are senior to you and also people that may be more junior. Email them ahead of time and schedule meetings during coffee or lunch breaks early in the conference.
- Do your homework before approaching a specific scientist. If you have some knowledge of their research, then a simple introduction can be had through a compliment or question about a specific piece of work. All scientists love to discuss their research, so if you have a question or insight to share they almost always want to hear it.
- Find an appropriate time to approach someone and be polite. A good time to introduce yourself might be at a reception or poster session; another meeting can always happen after the initial introduction.
- Go to poster sessions, or better yet, present a poster. Poster sessions are a great networking opportunity.
- Go to after program events (e.g. dinner, drinks and hikes). The best networking happens off campus.
- Ask a mentor, or another scientist who knows the researcher you would like to meet to introduce you and help break the ice.

## Writing Fellowship or Grant Proposals

Writing good fellowship and grant proposals is something of an art form in itself. As an early/mid career researcher writing your first proposal, it is easy to feel bewildered, not knowing quite where to start. It is hoped that the following guidance will set you in the right direction.

- Know your audience. Grant proposals are diverse and depend on the specific call in regard to what is required, what the review procedures will be and who will be the reviewers; therefore, always read the specific call/request for proposals carefully, so that you know what is expected and what the deadlines are.
- If appropriate, discuss your proposal with the specific program officer / agency’s program manager, if there is one, to be certain that what you are proposing fits the guidelines for support. They can often give good advice on what will be received well versus what will not be. Ask if the proposal will be reviewed by more than one group.
- Follow faithfully any guidelines that are given by the funding body, e.g. if you are asked to write the proposal in 12pt Arial font.
- Ask a successful grant writer to share some of their previous grants—the structure and level of detail as well as visual support for a proposal varies greatly and needs to be tailored to the specific call.
- Collaborate with someone who has been successful in obtaining support in the past from the agency.
- Try to plan ahead so that you have time to share a draft of your proposal with your peers or mentor for feedback.
- Make sure you have an exciting and innovative idea in the first place! Remember that the person(s) reviewing your fellowship application / grant proposal will probably have many others as well, so it is important to ‘grab the reviewer’s attention’ from the outset. Aim to write a factual but stimulating first paragraph which will make the reviewer want to read on and find out about the exciting project you are proposing.
- Ensure also that your idea is appropriate, carefully stating the goals of the proposed work somewhere near the start of the proposal.
- Write passionately from the heart and be ‘achievably ambitious’.
- Justify any claims you make and give as good an argument as you can that what you are about to do can be achieved.
- Do not try to cram every possible thing you can think of into the proposal; rather, be focused and have a good timeline with appropriate milestones.
- Most grants are scored badly because the reviewer could not understand what you really wanted to do. Far fewer fail because of a flawed idea, so make a big effort in articulating your ideas as clearly as possible; visual support can really help e.g. cartoons, schematics and graphs.
- Emphasize why you are the appropriate person to do the work.
- Almost nobody is successful with the first iteration of a grant, so it is good to submit to a call on the first round and then resubmit on subsequent rounds, integrating reviewer feedback.

For more on this topic, see ‘Notes on Writing and Getting Grants’ by Lou Gross: lgross.utk.edu/grantwriting.txt.

## Applying for Faculty Positions

Many early/mid career researchers may be relatively inexperienced in writing job applications, or be unsure of how best to present themselves to potential employers. The following advice is given with faculty applications in mind, though many of the tips are also relevant to applications for postdoctoral positions.

- Do not apply for a job you do not want—you might get it.
- Publish your work when it is ripe, even if it is not perfect.
- Collaborate, but be sure to establish your own identity.
- Think about who you are: a fox or a hedgehog? This reference comes from a 1953 book by the philosopher Isaiah Berlin, in which he quotes the Ancient Greek poet Archilochus as saying that ‘*the fox knows many things, but the hedgehog knows one big thing*’ (Berlin 1953). In the context of mathematical biology, think about whether you see your research as centring around one topic, or as touching on many topics, perhaps with a more abstract common theme. Both are valid ways to work, but it is good to think about who you are, to avoid getting pushed or pulled in directions that might not fit.

**CV**

- Make sure your CV is up-to-date and is written well.
- Do not try to exaggerate anything, e.g. do not list lots of unpublished papers.

**Cover letter**

- Read the job advertisement carefully and write a relevant, engaging cover letter, outlining your background, your current research interests, your future research plans and your teaching philosophy / teaching experience.
- Be explicit as to why you are appropriate for the position. Spend some time finding out about the department and the university in general, and aim to include in your cover letter how you feel you could fit in and connect with the teaching and research that is going on in the department and also potential collaborations elsewhere in the university (e.g. departments of biology / life sciences and medicine).
- Be enthusiastic.

**Statement of research interests**

- Summarize in one paragraph the main results from your prior work.
- Lay out a research plan, possibly with several different components. Think of this as a research plan for the initial 5–10 years of your career. Where do you want to be, what ‘big’ questions do you want to work on and how do the smaller ones fit into this?

**Statement of teaching interests**

- Summarize what your teaching experience has been.
- Give a bit of your teaching philosophy and provide examples of how you have applied it (e.g. projects you developed/used in a course you taught, or implementation of computer-based examples).
- State your teaching objectives over the next 5 years—what courses and seminars you might like to teach/develop, what texts you might be interested in developing. Tie this in to particular courses the university provides.

**Referees**

- Make sure you have good referees who will provide strongly supportive but not hyperbolic references.
- Make it as easy as possible for your referees to write a letter for you—give them all the material you are sending out, explaining how to address letters and providing the links to the adverts for positions you are applying to.
- Make sure the referees know which jobs are the ones of most interest to you.
- Perhaps ask your referees to contact (email or phone) anyone they know at your top choice positions to alert them to your application.

For more on this topic, see ‘Applying for a job, haggling for a job, and keeping a job’ by Lou Gross: lgross.utk.edu/gettingjobs.postdocs.mbi06.txt.

## Preparing and Giving Lectures

The average early/mid career researcher will have attended hundreds of lectures during their undergraduate studies; some of them better than others. While many PhD students will get experience of leading or assisting with tutorials and problems classes, opportunities for lecturing experience arise less frequently. The following guidance should be of help to postdoctoral researchers and new faculty preparing to give their first lectures.

- Find the lecturing style that you are most comfortable with e.g. ‘chalk and talk’, slides, iPad/Tablet etc., and practise at it.
- Do not practise too much—talks can sound really canned with too much practice. Put another way, too much practice can stand in the way of ‘presence’ during a talk, thinking a little on your feet and taking a few chances.
- Prepare your notes in advance and try to connect with external material e.g. books, research articles, online videos etc.
- Think about your main point during your pre-lecture preparation.
- Your lecture has to fit your audience. Do not attempt to give the same lecture to biologists and to mathematicians.
- Optimise your slides: a maximum of 20 words per slide, brief bullet points, self-contained and easy to follow.
- Do not include something on a slide if you do not want to talk about it.
- Go to the lecture theatre before you start the course and work out where everything is so that you can begin the first lecture without any glitches or delays.
- Try to be enthusiastic and passionate in your delivery and to ENJOY giving the lecture.
- Never forget that it is about the material, not about you.
- Consider introducing your talk with interesting scientific questions, and returning to those at the end to show that you ‘solved them’. Merely reproducing a behaviour with a model is not very interesting unless you can show new insights or novel predictions.
- Aim to engage the students rather than just lecture for one hour e.g. stop regularly and ask questions, ask the students to suggest ways to complete a piece of algebra or offer the answer to a problem.
- Provide plenty of motivation and background for the audience to understand the main ideas. Be sure to emphasize the significance and goals.
- Give plenty of worked examples in the class which underpin any piece of theory you deliver.
- Be sure to EXPLAIN everything. Your audience will appreciate that.
- Make the lecture interesting. Use some colour, make fonts nice and large, consider some humour if possible, once you gain confidence.
- Make a deliberate mistake now and again—this can encourage the students to engage and when they get the correct answer it gives them confidence. It also shows them that you are not infallible!
- Never go over time.

## What Do You Wish You Had Known When You were an Early-mid Career Researcher?

In addition to asking our seasoned professionals for guidance on specific questions, their advice to early and mid career researchers was also sought at a more general level, as recounted in this section and that which follows. First, in this section, we explore the hard-earned knowledge that our experts wish they had possessed when they were early/mid career researchers.

**Seeking advice**

- Do not be shy about getting advice, particularly on grant proposals.
- Understand how the system at your institution works, who to go to for advice/assistance and how to work around arcane rules that constrain your ability to advance your research and teaching.

**Career planning**

- Think a few years ahead but do not let long-term planning stand in the way.
- Early in your career, it is common not to know what you really want and that is OK, since you have not experienced enough yet.
- ‘When I started as a graduate student, I had a very specific plans about what I wanted to study: quantum chemistry. Like most mathematical biologists, I never intended to be one! I stumbled onto the field through my professors and mentors. So keep your eyes open, see what catches your interest, see where new research areas are opening up and where you can make a contribution. Be flexible, find your place in the world and have fun!’
- Think strategically about what you will gain from a specific position and how it might lead you to new opportunities in the future.

**‘Failure’ and rejections**

- Be ready to accept rejections and how to move on effectively from these, such as re-applying for grants to either the same agency which initially rejected it or to try someplace else.
- Do not take failure personally; academia is a constant source of failure, whether it is papers, grants or even your science. Failure is the only way we can learn; of course it still stings, but know that this is a universal pain we all feel as scientists, and it is also temporary, as it will drive resubmissions, rewriting, reframing and ultimately success.
- Lack of a job offer, or interview, may just be due to various political factors in a given department/unit that have nothing to do with your excellence. Therefore, do not let such ‘rejections’ affect your morale and work.

**Research**

- Do not skip your postdoc; exploit every second of it. It is a rare time in your scientific career that you will never have again—both scientific freedom and no financial concerns.
- Have fun! Most research ideas come outside the laboratory; on a walk, while exercising, or while having dinner with friends. A lot of great ideas start out on a napkin.
- If you are not excited about a problem, the work is not going to be worthwhile.
- You can work on anything you want to, independent of your field, as long as you are willing to learn the new area.
- Keep doing good work, even when the job-market looks bleak. Eventually this will pay off.
- Take the time to learn new skills.
- Do the hard work yourself.

**Skills**

‘I wish I had known’:

- LaTeX —‘I wrote my PhD thesis using troff’ (wikipedia.org/wiki/Troff).
- More numerical analysis.
- The Sobolev Embedding Theorem (just kidding!).

**Sharing your work**

- Put real effort into making your science as accessible as possible—the more people who understand it, the better it will be cited and shared.
- Grab any opportunity you can to present your work, even if you find it difficult. It will help you understand your own work better and expose the community to what you are doing and critically provide valuable feedback.
- Open science is a golden opportunity to share your work before it is published, embrace it. Share your papers on preprint servers (e.g. bioRxiv and arXiv), and your code and data on public repositories (e.g. GitHub).

**Collaboration and networking**

- Work with people you like, in labs that are happy and have a good community ethic. Do not try to work with people simply because of their prestige.
- Use administrative roles to build collaborations.
- Networking with others (in your unit and at conferences) is very important. Consider sending your e-publications to the top researchers with a short email. Many are busy, so may not answer, but some will.
- Do not be shy at a conference. Schedule meetings ahead of time to make sure you are not alone during coffee breaks.
- Maintain contact with those you have met who might help your career advance in the future.

**Organising your time**

- ‘I did not realize how much time I would spend in service-related activities. I sit on many committees. My service takes about one full day per week.’
- ‘I did not realize how much grant writing I would be doing. I had to learn how to write grants for many different reviewing bodies. This can take time, but can also be helpful in that you then understand how to talk about your research with many different audiences.’
- Teaching and mentorship activities can occupy much of your time. Make sure that you structure your week so that you have research blocks that are long enough for you to remember what you are doing, and get some work done to review and advance your projects.
- Get home in time for dinner with your family.

## Some Final Words of Advice

In this last section, we offer some final words of advice, not covered by the previous sections.

**Community, collaboration and care**

- Collaborate broadly and build your network of collaborators in ways that stretch your research to fields that might be far from your formal education.
- Team science is truly a gift for mathematical biology. It is being embraced across many different disciplines and is a golden opportunity to work across fields with creative teams, where the team is far more powerful scientifically than any of the individuals. If you can work with a team, jump at the chance.
- Develop a community around you, but do not feel that you need to collaborate with everyone. Deliberately keep some experts in your field at ‘arm’s-length’ as you will need people to review your file at tenure and promotion, for grants, and your manuscripts for publication.
- Care about your community—take time to contribute, to nurture and enrich your community as it will not continue without it.
- Make time for self-care; something outside of science even if it is with scientists. It is important to recharge your creative and non-creative batteries and that cannot happen if you use them all the time.
- Most scientists are good people even if they may ask difficult questions and appear intimidating—they are a scientist just like you and care about similar things.
- Always be honest, even if it means admitting mistakes, being truthful will always pay dividends in the end.

**Research**

- Work on what you want, not on what other people think you should.
- Enjoy yourself, have fun, work on problems that you are really interested in and passionate about.
- Aim high. Always ask ‘could my work be better?’ Do not settle for the first result and hurry to publish—do your due diligence and make that sure every piece of work has the highest impact it can.
- Mathematical biology is a subfield of biology. Talk to biologists as often as you can. Let their questions guide your research.
- Do not be afraid of data. Indeed, look at the data! You may find something that you did not expect that is more interesting than what you did expect.
- Understand what it means to calibrate and validate a mathematical model. Not every curve that fits data makes a model plausible and it does not guarantee predictive power (if that is what you are aiming for).
- Do not be a one-trick pony. It will help your career if you become the go-to person in the world on a particular topic, but do not constrain yourself to this area. Look for side-projects that may be well outside this area of focus.
- Be willing to take risks and try out new/alternative things. It is only by failing that we discover what does not work and this helps put us on another track that perhaps will work. Do not be afraid to ‘fail’. The following quote from John Backus (who invented FORTRAN) illustrates this point:
  ‘*I, myself, have had many failures and I’ve learned that if you are not failing a lot, you are probably not being as creative as you could be—you aren’t stretching your imagination. You need the willingness to fail all the time. You have to generate many ideas and then you have to work very hard only to discover that they don’t work. And you keep doing that over and over until you find one that does work.*’ —mathshistory.st-andrews.ac.uk/Biographies/Backus/quotations/

**Communication**

- Learning to communicate in writing and orally is just as important as doing advanced research. Your funding and the respect you achieve will depend on your ability to explain your work and convince others that it is significant.
- Work to build your vocabulary to be able to communicate with experts in fields quite different from your own.
- Get some formal training from science communication experts to assist you in being able to discuss your work with non-scientists and journalists. Do not be bashful about tooting your own horn.

For more on careers in academia, see ‘Careers in Academia: How to Enhance your Chances for Success’ by Lou Gross: lgross.utk.edu/eeb504Spring2021.html.
